# Computational Evaluation of Surgical Design for Multisegmental Complex Congenital Tracheal Stenosis

**DOI:** 10.1155/2020/3509814

**Published:** 2020-04-20

**Authors:** Limin Zhu, Xiaolei Gong, Jinlong Liu, Youjin Li, Yumin Zhong, Juanya Shen, Zhuoming Xu

**Affiliations:** ^1^Department of Cardiothoracic Surgery, Shanghai Children's Medical Center, Shanghai Jiao Tong University School of Medicine, Shanghai 200127, China; ^2^Institute of Pediatric Translational Medicine, Shanghai Children's Medical Center, Shanghai Jiao Tong University School of Medicine, Shanghai 200127, China; ^3^Shanghai Engineering Research Center of Virtual Reality of Structural Heart Disease, Shanghai Children's Medical Center, Shanghai Jiao Tong University School of Medicine, Shanghai 200127, China; ^4^Department of Otorhinolaryngology-Head & Neck Surgery, Shanghai Children's Medical Center, Shanghai Jiao Tong University School of Medicine, Shanghai 200127, China; ^5^Department of Radiology, Shanghai Children's Medical Center, Shanghai Jiao Tong University School of Medicine, Shanghai 200127, China

## Abstract

Multisegmental complex congenital tracheal stenosis (CTS) is an uncommon but potentially life-threatening malformation of the airway. Staged surgery is indicated for the complex pathophysiology of the abnormal trachea. Surgical intervention to fix the stenotic segments may result in different postoperative outcomes. However, only few studies reported the design of surgical correction for multisegmental CTS. We used computer-aided design (CAD) to simulate surgical correction under different schemes to develop a patient-specific tracheal model with two segmental stenoses. Computational fluid dynamics (CFD) was used to compare the outcomes of different designs. Aerodynamic parameters of the trachea were evaluated. An obvious interaction was found between the two segments of stenosis in different surgical designs. The surgical corrective order of stenotic segments greatly affected the aerodynamic parameters and turbulence flows downstream of tracheal stenosis and upstream of the bronchus. Patient-specific studies using CAD and CFD minimize the risk of staged surgical correction and facilitate quantitative evaluation of surgical design for multiple segments of complex CTS.

## 1. Introduction

Congenital tracheal stenosis (CTS) is defined as structural obstruction of the airway and is frequently associated with cardiovascular malformations. Moderate and severe types of CTS according to Anton-Pacheco's functional classification are characterized by varying degrees of respiratory symptoms and life-threatening respiratory conditions in critical cases [1–3]. Due to the high mortality, surgical intervention has been regarded as the best option for young infants with severe respiratory symptoms. The surgical techniques based on the nature of CTS include resection of the stenotic segment with end-to-end anastomosis in short-segment CTS, slide tracheoplasty, and patch tracheoplasty in long-segment CTS [3–6]. Although the above surgical interventions facilitated correction of the complex CTS, the inherent limitations of surgeries may still lead to complications and defective prognosis [7]. Primary complete repair should be at high risk of complication and mortality for the complex CTS patient; the palliative staged procedure will be considered.

Increased duration of cardiopulmonary bypass (CPB) is the major risk factor for mortality in neonates and young infants. Therefore, surgical strategies for CTS mainly focus on releasing tracheal stenosis in respiratory distress as well as reducing the duration of CPB [3]. Simultaneous resection of multiple stenoses and primary anastomosis leads to excessive tension, which affects the vitality of suture sites and induces delayed granulation or restenosis [7]. Therefore, staged repair is usually required in complex cases of multiple segmental CTS to prevent excessive tension and shorten the duration of CPB. Although several studies showed acceptable results of tracheoplasty, the surgical design to realign the stenotic segments may result in different postoperative outcomes. The late mortality ranges between 5.5% and 12%, and more than 20% of the surviving cases need reintervention postoperatively [8–10]. The surgical scheme to rectify stenosis may result in different postoperative courses and treatment outcomes. It still remains a great clinical challenge since limited aerodynamic features of the trachea are available for patient-specific treatment [2, 5].

Recent improvements in medical imaging and computational fluid dynamics (CFD) have facilitated the study of patient-specific complex CTS in three dimensions by evaluating the aerodynamic characteristics. Airflow simulations were conducted using pressure and velocity in tracheal geometries reconstructed from medical images. The results provide quantitative data, such as pressure drop, energy loss (EL), and wall shear stress (WSS) that are not easily available through direct measurements in a clinical practice. The CFD approach allows objective and quantitative evaluation of CTS [2]. Several studies investigated tracheal aerodynamics in recent years [2, 11–18]. Brouns et al. discussed the effects of different ratios and lengths of stenosis on pressure drops in a simplified tracheal model [11]. The pressure drop at rest was only affected in case of severe constriction, suggesting that the simulated flow dependence was a means of detecting stenosis at the precritical stage. Mimouni-Benabu et al. performed steady calculations using CFD based on patient-specific models across a wide range of ages and stenosis classifications [2]. They successfully validated the results with clinical measurements of pressure drops. Ho et al. utilized CFD to study the pressure drop after implantation of a tracheal stent and found that the pressure drop decreased significantly, which was consistent with clinical measurements of pulmonary function [13]. All these studies indicated that CFD was appropriate to determine the regional aerodynamics of the tracheal stenosis in patient-specific studies. It is a promising strategy for the aerodynamic evaluation of different designs of staged surgical correction for complex CTS.

In the present study, we conducted a patient-specific investigation based on the analysis of medical images. A complex CTS model with two stenotic segments was constructed. Computer-aided design (CAD) was used to create new tracheal models virtually. CFD facilitated the evaluation of the outcomes under different schemes. Pressure drops, WSS, airflow distribution, and EL rate were calculated to estimate the local tracheal aerodynamics. The aim of this study was not only to disclose the local aerodynamic features in the two segmental stenoses for prediction of surgical outcomes but also to perform virtual design and quantitative evaluation of patient-specific surgical correction for the staged correction of complex CTS with multiple stenoses.

## 2. Materials and Methods

### 2.1. Patient Information and Geometric Model Generation

Clinical studies were conducted in a 5-month-old baby following informed consent of the baby's parents. Protocols were approved by the local institutional review board and regional research ethics committee of Shanghai Children's Medical Center (SCMC) Affiliated Shanghai Jiao Tong University School of Medicine.

The 5-month-old male was hospitalized in the pediatric intensive care unit due to severe pneumonia and respiratory failure. He developed obvious respiratory distress and hypercapnia although he was managed with mechanical ventilation immediately after hospital admission. He was diagnosed with left pulmonary artery sling (PA-Sling), two stenotic segments in the trachea by echocardiography and CT-scan.  Table 1 displays the patient data.

**Figure 1:**
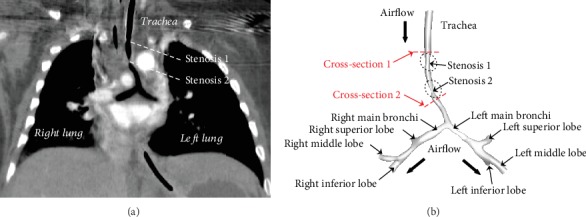
CT image and three-dimensional (3D) reconstruction: (a) the analysis of patient-specific CT image; (b) reconstruction of 3D tracheal geometry with main bronchi from original CT images.

To obtain patient-specific computed tomography (CT) images for the 3D geometric generation of the tracheal model, a series of continuous 0.625 mm thick CT images of the thorax were acquired by a 16-slice multidetector row enhanced CT scanner (BrightSpeed Elite, GE Medical System, General Electric, America). Medical imagining software Materialise® Mimics 19.0 was used to compile and reconstruct the 3D tracheal geometry. To reconstruct lumenal geometry, a window level value (WL, 180 Hu) was used to refine 3D geometry. The accuracy of the reconstructed model was checked against the geometry with an exact measurement in original DICOM CT slice files.  Figure 1 depicts the geometry after surface smoothing with the trachea and main bronchi. Two stenoses including stenosis 1 (S1) and stenosis 2 (S2) were observed clearly in the 3D patient-specific model. We defined the patient-specific model as Model 1 (M1) and exported it in stereolithography interface format (STL) that was used as the geometry input format in most CAD software for virtual design of different surgical schemes.

To evaluate the degree of stenosis, we defined the stenosis ratio (*r*) mathematically as follows:

(1) $$\begin{array}{c} r=\frac{S_{\text{TE}}-S_{\text{SP}}}{S_{\text{TE}}}\times 100\%, \end{array}$$

where *S*_TE_ represents the cross-sectional area of the tracheal entrance and *S*_SP_ denotes the minimal cross-sectional area of the stenosis. Based on this definition, a stenosis ratio of 68.7% and 86.5% was found in the patient-specific model at S1 and S2, respectively.

In the patient's original model, the stenotic part, between the two cross-sections of the stenosis, was more than 30% of the whole trachea in length and less than the normal lumen of the trachea in diameter. Based on the morphological classification of CTS, the patient-specific trachea represents generalized narrowing with two stenotic segments, instead of pure segmental or diffuse stenosis. Due to the relatively large pathologic region, a long duration of cardiopulmonary bypass time is needed to correct two stenoses simultaneously, which increases the risk factor of surgical mortality in a critical young infant. Therefore, staged surgeries are required for the correction of both the stenoses. The initial decision is critical for the determination of surgical corrective order of the two stenoses to avoid adverse events associated with residual stenosis or the risk of leakage and restenosis caused by excessive tension at anastomosis sites [1]. Accordingly, two possible staged surgical schemes for stenosis corrections were designed.

**Figure 2:**
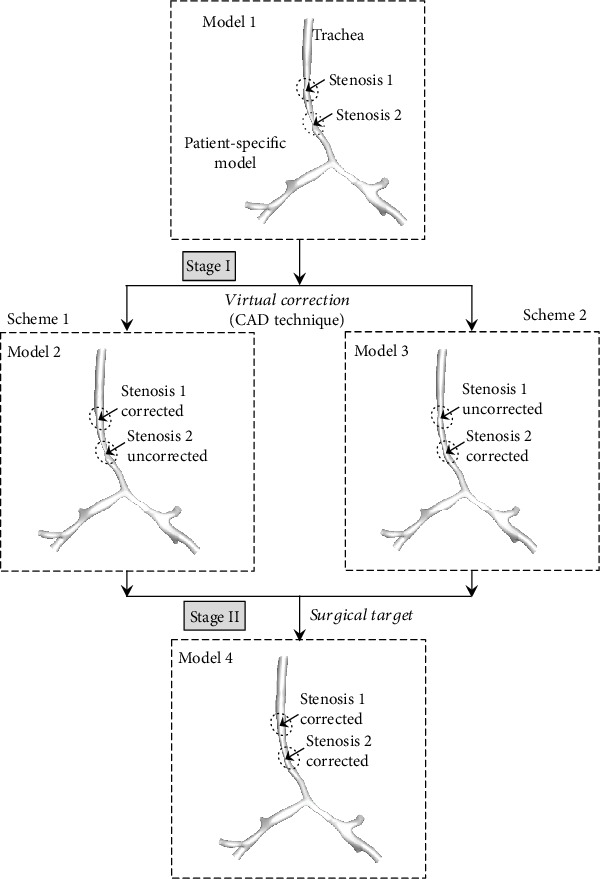
Two surgical schemes virtually designed by the approach of computer-aided design (CAD) for the patient-specific tracheal model with two segmental stenoses.

Based on the M1, we performed virtual operations and aerodynamic analysis to determine the surgical scheme for the correction of complex CTS with two tracheal stenoses. Model rebuilding was carried out around the areas of the stenosis to virtually simulate surgical outcomes of different schemes. The CAD software Materialise® 3-matic 11.0 was used to modify the original stenotic segment to normal size based on the analysis of patient-specific CT images. Three new models, Model 2 (M2), Model 3 (M3), and Model 4 (M4), were created. In Figure 2, the two possible staged surgical schemes are described.

### 2.2. Airflow Analysis

The tracheal airflow is defined by a 3D incompressible Navier-Stokes (N-S) equation and a continuity equation. The airflow motion was described by the following equations that describe the most general movement of fluid medium:

(2) $$\begin{array}{c} \left\{\begin{array}{c} \frac{\partial }{\partial t}\left(\rho \;u_{i}\right)+\frac{\partial }{\partial x_{j}}\left(\rho \;u_{i}\;u_{j}\right)=-\frac{\partial p}{\partial x_{i}}+\frac{\partial }{\partial x_{j}}\left[\mu \left(\frac{\partial u_{i}}{\partial x_{j}}+\frac{\partial u_{j}}{\partial x_{i}}\right)\right]+f_{i},\\ \frac{\partial \rho }{\partial t}+\frac{\partial }{\partial x_{j}}\left(\rho \;u_{j}\right)=0, \end{array}\right. \end{array}$$

where *i*, *j* = 1, 2, 3; *x*_1_,  *x*_2_,  and *x*_3_ represent coordinate axes; *u*_*i*_,  *u*_*j*_, and *p* denote the velocity vectors and the pressure in the fluid domain; *ρ* and *μ* are airflow density and viscosity, and *t* is time. The term *f*_*i*_ expresses the action of body forces.

Due to the presence of mucous cells along the trachea, water vapor should be increased during the airflow. However, during mechanical ventilation, air is humidified by body temperature in the clinical practice. Dehydration of the mucous cells is rare. Therefore, the components of airflow in the trachea are relatively stable. The air density is a constant. Due to mechanical ventilation to generate steady airflow, the air in the trachea should meet the requirements of Newtonian fluid. We assumed airflow to be a Newtonian fluid with a constant density (*ρ* = 1.161 kg/m^3^) and viscosity (*μ* = 1.864 × 10^−5^ kg/m s), and the body forces were omitted [2, 13].

The Reynolds number (Re), the ratio of the inertial forces to the viscous forces in the flow, is defined by

(3) Re=ρ UDμ,

where *U* and *D* denote the characteristic fluid speed and the length scale, respectively. We calculated the Reynolds number based on the flow at S1 and S2. We found a maximum value of about 3500. According to the theory of stenotic flow, a region of high velocity described as jet flow occurred downstream of a major stenosis. Transition in a steady jet occurs at a Reynolds number of approximately 1000, and fully turbulent flow appears at 3000 [19]. Therefore, the airflow in this patient-specific tracheal geometry was characterized by turbulence.

To address the problem of turbulence, we used the Wilcox *k*‐*ω* model, which was validated perfectly for the complex airflow in the trachea [20]. The Wilcox *k*‐*ω* model is a commonly used two-equation eddy viscosity model based on the equations for the kinetic energy of turbulence (Equation (5)) and the specific dissipation rate (Equation (6)) defined below:

1. Eddy viscosity:

(4) $$\begin{array}{c} \mu_{T}=\rho \frac{k}{\omega } \end{array}$$

(2) Turbulence kinetic energy:

(5) $$\begin{array}{c} \rho \frac{\partial k}{\partial t}+\rho u_{j}\frac{\partial k}{\partial x_{j}}=\tau_{ij}\frac{\partial u_{i}}{\partial x_{j}}-\beta^{*}\rho k\omega +\frac{\partial }{\partial x_{j}}\left[\left(\mu +\sigma^{*}\mu_{T}\right)\frac{\partial k}{\partial x_{j}}\right] \end{array}$$

(3) Specific dissipation rate:

(6) $$\begin{array}{c} \rho \frac{\partial \omega }{\partial t}+\rho u_{j}\frac{\partial \omega }{\partial x_{j}}=\alpha \frac{\omega }{k}\tau_{ij}\frac{\partial u_{i}}{\partial x_{j}}-{\beta \rho \omega }^{2}+\frac{\partial }{\partial x_{j}}\left[\left(\mu +{\sigma \mu }_{T}\right)\frac{\partial \omega }{\partial x_{j}}\right] \end{array}$$

(4) Closure coefficients:

(7) $$\begin{array}{c} \alpha =\frac{5}{9},\\ \beta =\frac{3}{40},\\ \beta^{*}=\frac{9}{100},\\ \sigma =\frac{1}{2},\\ \sigma^{*}=\frac{1}{2}, \end{array}$$

where *τ*_*ij*_ is the Reynolds stress tensor. It is given by

(8) τij=2μTS−ij23ρkδij,

where *S*_*ij*_ denotes the mean strain-rate tensor and

(9) $$\begin{array}{c} \delta_{ij}=\left\{\begin{array}{cc} 1, & \text{if }i=j\\ 0, & \text{if }i\neq j \end{array}\right\} \end{array}$$

is Kronecker delta.

### 2.3. Aerodynamic Parameters

Incompressible steady flow through a stenosis exhibits a characteristic pattern. Pressure drop, the difference of static pressure between the upstream and downstream flows across the narrowed area of the trachea, creates a high velocity jet of air. In this case, turbulence with a high Reynolds number proximal to the constriction occurred instead of low velocity converging flows. Subsequently, flow disruption and energy losses were generated, which may increase the airway resistance and lead to ventilatory difficulty. The severity of stenosis can be estimated by pressure drop.

WSS, which is difficult to measure directly, is a manifestation of the interaction between the fluid and its solid boundary. The equation for WSS symbolized by *τ*_wall_ in a Newtonian fluid is expressed as follows:

(10) $$\begin{array}{c} \tau_{\text{wall}}={\left.-\mu \frac{\partial u_{x}}{\partial y}\right|}_{y=0}, \end{array}$$

where *μ* represents viscosity, *u*_*x*_ denotes the velocity of the fluid near the boundary, and *y* is the height above the boundary. When WSS is high, the effects of shear stress on the boundary wall induce tracheal damage [21, 22].

EL, the energy difference between the tracheal inlet and the outlet, is useful for the evaluation of respiratory resistance. Based on the pressure and flow rate of the inflow and outflow, EL is calculated by

(11) $$\begin{array}{c} \text{EL}=E_{\text{inlet}}-E_{\text{outlet}}=\underset{\text{inlet}}{\sum }\left(P_{\text{i}}+\frac{1}{2}\rho u_{\text{i}}^{2}\right)\;Q_{\text{i}}-\underset{\text{outlet}}{\sum }\left(P_{\text{o}}+\frac{1}{2}\rho u_{\text{o}}^{2}\right)\;Q_{\text{o}}, \end{array}$$

where *P* represents the static pressure, *u* denotes the velocity, and *Q* is the flow rate. The term i indicates the inlet at the trachea and o indicates the outlet at the left main bronchi (LMB), left superior lobe (LSL), left middle lobe (LML), left inferior lobe (LIL), right superior lobe (RSL), right middle lobe (RML), and right inferior lobe (RIL). In order to evaluate the effects of stenosis on EL, we defined the energy loss rate (*λ*) as a normalized factor, which was described by

(12) $$\begin{array}{c} \lambda =\frac{\text{EL}}{E_{\text{inlet}}}=1-\frac{E_{\text{outlet}}}{E_{\text{inlet}}}. \end{array}$$

### 2.4. CFD Analysis

#### 2.4.1. Mesh Generation

**Figure 3:**
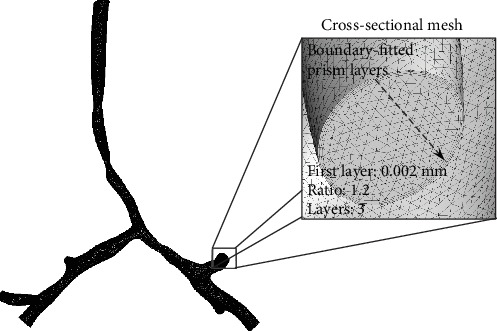
Mesh information of each model used in CFD simulation.

The grid-generation software, ANSYS® ICEM 14.0, was used to produce mixed grids. Three-layer body-fitted prismatic grids were created in the near-wall regions with an average nodal space that increased by a ratio of 1.2. The distance of the first prismatic layer to the tracheal surface was fixed at 0.0022 mm. This scheme accurately measured WSS and improved the resolution of the relevant scales in fluid motion. A tetrahedral mesh covered the remainder of the domain.  Figure 3 provides detailed information of the mesh.

To determine the best mesh for CFD analysis, grid-sensitivity verification was performed. We found that grid numbers of about 0.5 million in steady simulation created the most efficient mesh.  Figure 3 and Table 2 show the mesh information for each model used in the present study.

#### 2.4.2. Boundary Conditions

In the present study, the patient was an infant with respiratory failure resulting from complex tracheal stenosis. A ventilator was used before the surgical corrections for CTS due to respiratory distress in this patient. Although the ventilator provides several flow patterns, such as constant flow in volume control, flow in pressure control, and the sine wave, the intrinsic positive end expiratory pressure (PEEP) was high when the patient was ventilated. It was difficult to manage mechanical ventilation. Therefore, we used the constant flow in volume control during mechanical ventilation and maintained the paralyzed patient with muscle relaxants in the clinic. Based on the patient-specific treatment conditions, our simulation was conducted using constant flow. The boundary conditions in the simulation were identical to the reality of mechanical ventilation. Constant airflow was delivered into the lungs with the volume control of 3 L/min. Accordingly, we simulated steady flow and adjusted the inlet mass flow to the same value.

To fully develop the flow boundary layer, we extended the inlet domain upstream 20-fold compared with the size of the trachea. At the outlet, airway was extended forty times the diameter in a normal direction to facilitate sufficient recovery of air pressure in each branch. A zero pressure was assumed at the outlets according to Ho et al. [13].

#### 2.4.3. Calculation

The finite volume solver package, ANSYS® FLUENT 14.0, was used to solve the steady airflow in each model. We assumed that the tracheal wall consisted of rigid surfaces with no-slip conditions. The semi-implicit (SIMPLE) method was selected to solve the discretized 3D incompressible N-S equations. A second-order upwind scheme was used. For convergence criteria, the relative variation of the quantities between two successive iterations was smaller than the preassigned maximum, 10^−5^. All the calculations were performed on a computer workstation with a 64-bit Windows 7 operating system. The workstation was equipped with double CPUs: Intel (R) Xeon X5690 3.46 GHz processors with 24.0 GB RAM memory.

## 3. Results

**Figure 4:**
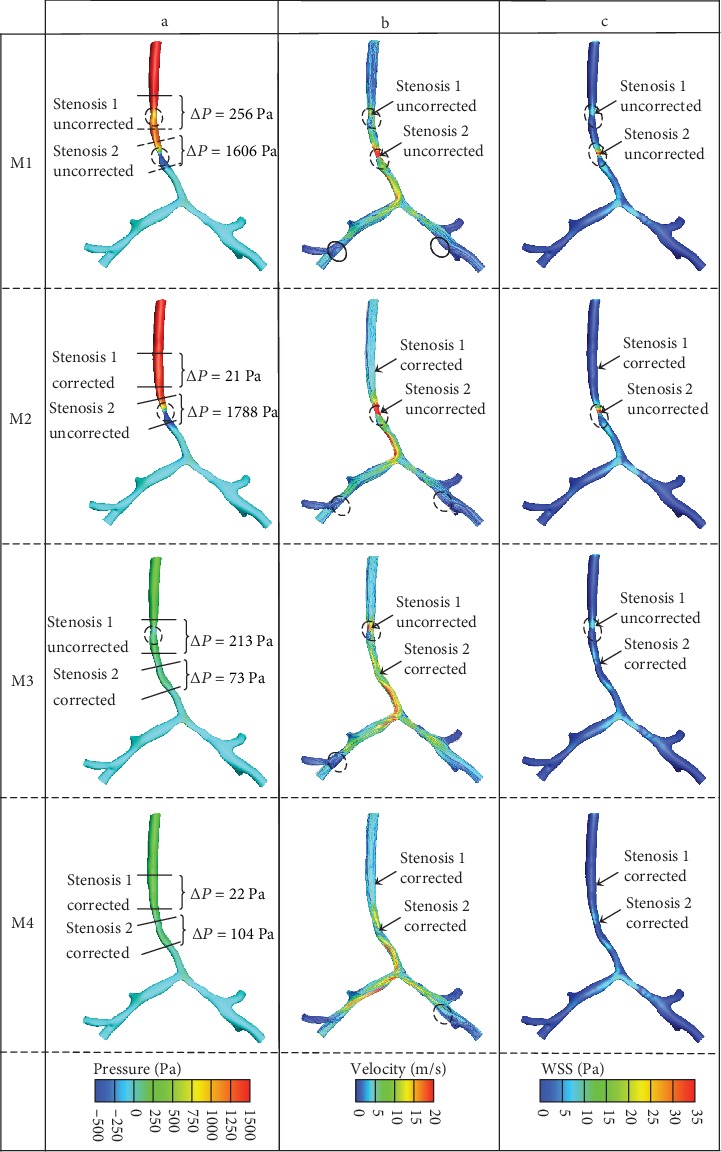
The contour plots of local aerodynamic parameters: (a) pressure drop; (b) streamlines. Broken circle indicates reverse flow. (c) Wall shear stress.

The constriction ratios at S1 and S2 were 68.7% and 86.5% in M1, respectively. We calculated pressure drops around the area of S1 and S2 in the four models.  Figure 4(a) shows the results on the contour plots of total pressure. Compared with these models, an obvious interaction was found between the two segments of stenosis. In M2, the correction of S1 increased the pressure drop at S2. On the other hand, the correction of S2 decreased the pressure drop at S1 in M3. In the original M1, pressure drop was much higher at S2 than at S1. When both segments of stenosis were corrected in M4, a little pressure drop still remained at previous stenotic sites. However, M4 is an ideal surgical model, which is not feasible in young infants, because of the severity before operation. The clinician should select a better option to improve the symptoms with minimal secondary organ damage.

Figure 4(b) shows the streamlined pattern of the four models. Turbulence flows occurred at the downstream regions of the two segments of tracheal stenosis and the upstream regions of bifurcation in the right middle bronchi (RMB) and right inferior bronchi (RIB) and LMB and left inferior bronchi (LIB). Two turbulence flows were observed downstream of S1 and S2 in M1. After correction of stenosis, the downstream turbulence flow was eliminated in M2 and M3, respectively. None of the turbulence flows was found in M4, when all the stenoses were corrected. Compared with the four models, we found that the turbulence flow downstream of S1 and S2 affected the turbulence flow upstream of the bifurcations in the RMB, RIB, LMB, and LIB. Correction of the stenoses reduced the scales of turbulence flow at these regions.

Figure 4(c) displays high values of WSS in S1 and S2. Airflow through the trachea increases the velocity and gradient to the wall due to stenosis, which elevated WSS and reinforced the interaction between the airflow and the tracheal wall. It indicated loss of energy at this location. After correction of stenosis, WSS decreased significantly.  Table 3 lists the details of WSS at S1 and S2. Relatively high value of WSS is also observed at the bifurcation of the main bronchi. Combined with the analyses in Figure 4(b), the complex turbulence due to the distorted spatial configuration of the trachea might be one of the main factors.

**Figure 5:**
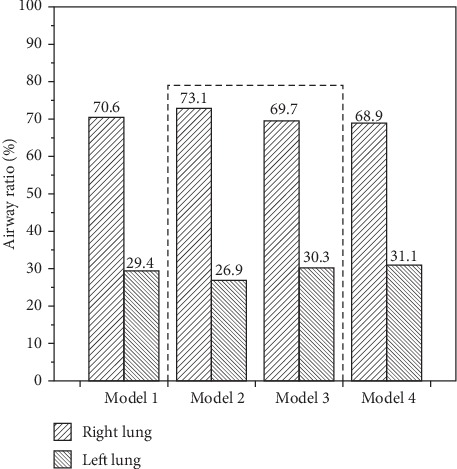
Airflow ratio distributed to two lungs.

The ratios of airflow distribution to the right and left lungs are calculated and compared in Figure 5. The ratios are very similar among the four models, close to 7 : 3, with no obvious differences following correction of stenoses. This result indicates that stenosis might not be the main factor underlying the altered airflow distribution ratio.

**Figure 6:**
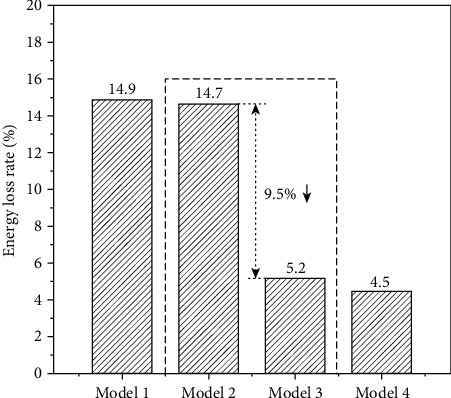
Comparison of energy loss rate.

The bar graph in Figure 6 describes the EL rate. In M1, the EL rate of the whole trachea with two stenoses is approximately 14.9% of the inlet energy. Similar results were found in M2 following S1 correction. However, a significant decline of the EL rate occurred when the S2 was corrected. The EL rate was approximately three times lower in M3 than in M1. A similarly low rate of approximately 4% was observed in M4. Similar improvements in CFD data of M3 compared with M4 suggest an easier option to improve the symptoms of respiratory distress in critically young infants.

## 4. Discussion

In the present study, we introduced the technique of CAD to create new tracheal models simulating different surgical designs. CFD was used to analyse local aerodynamics to predict postoperative outcomes comparing different surgical designs of stenosis correction. Aerodynamic parameters such as pressure drop, WSS, EL, airflow pattern, and the ratio of airflow distribution, which are not easily determined clinically, were calculated to determine the local aerodynamics around stenotic areas. The results indicated that an improved surgical design for the staged correction greatly affected the aerodynamic interactions between multiple stenoses. The correction of stenosis alleviates the increase in pressure drop, WSS, and EL.

The M4, the ideal surgical repair model, represents the final status after total surgical corrections of two stenoses in the patient-specific CTS model. However, the pressure drop still existed. Around the area of S2, the pressure drop was higher than that in M3. The pathological structure of the trachea may still exist in M4 although both stenoses were corrected.

After the correction of S1, which limited the airflow to S2, S2 was relatively more stenotic than S1 compared with M4 and M3. Comparing M1 to M3, a decline in pressure drop was observed at S1. Nevertheless, an increase in pressure drop at S2 was detected when M1 was compared with M2 suggesting interaction between the two parts of tracheal stenosis. Therefore, CTS with more than one stenosis could not be simply regarded as multiple segmental stenoses but a comprehensive lesion.

Turbulence flows were observed downstream of stenosis. The high-pressure difference across the stenotic area creates an increased jet flow. Adverse pressure gradient inhibits the flow. Turbulence flows occurred as the direction of velocity changed downstream of stenosis. Due to the distorted spatial configuration of the trachea and shift velocity, turbulence flows were developed when airflow encountered tracheal bifurcations of the RMB and RIB, and LMB and LIB. After the correction of stenosis, the shift of velocity was relieved and turbulence flows were decreased.

WSS was regarded as a measure of the interaction between flow and its solid boundary. Ramzi et al. discussed the effect of WSS created by the peak expiratory flow rates and found that high WSS induced additional trauma to an already inflamed epithelial layer of bronchi [22]. Chowdhary et al. disclosed that WSS exacerbated the damage of the bronchial epithelial layer [21]. Green confirmed the experimental findings using CFD to simulate the peak expiratory flow in the trachea and major lung bronchi [23]. He pointed out that the WSS (19 Pa) was high enough to damage the bronchial epithelial layer. In the present study, Table 3 compares the WSS at the stenosis before and after virtual correction. A significantly higher value of WSS was produced at S2 due to greater geometric restriction of the tracheal lumen. An obvious decline was observed following correction (Figure 4(c)). It is speculated that infants with CTS showed bronchial epithelial injury before surgical correction and early correction in severe cases may reduce the risk of additional trauma to the epithelial layer caused by high WSS.

There was no remarkable difference in the ratio of airflow distribution to two lungs in every model with or without virtual correction. In contrast, Ho et al. found that the percentage of airflow distribution was significantly improved after the implantation of the tracheal stent in adults [13]. Compared with the CTS in congenital cardiovascular anomalies, the varying morphology of the trachea in adults with cancers of the thyroid, esophagus, breast, and lung might be one of the main reasons. In healthy infants, the ratio of airflow distribution to the right and left lungs is approximately 56.57% : 43.43% [13]. However, the ratio is 68.9% : 31.1% in our patient-specific model after the two stenoses were corrected. Probably, the morphology of and the angle between the trachea and the main bronchi in this patient are still pathologically abnormal even though the stenotic segments were corrected. This phenomenon was further explained by the C-shaped trachea caused by the anomalous left pulmonary artery. The left bronchi can be reached more anatomically. It was consistent with findings in Qi's research [14] suggesting that the C-shaped trachea may facilitate a larger airflow into the left lung and decrease the ventilation of the right side.

The geometric restriction of stenosis altered the airflow direction and accelerated the local velocity, which sharply increased the pressure drop, WSS, and EL. The low pressure drop and EL reduce the respiratory resistance and improve ventilation. They play a key role subsequently in eliminating the risk factors of the trachea. EL was introduced to avoid the geometric effects in four models. A significant decline in the rate of EL occurred following the correction of severe stenosis, S2, as shown in Figure 6, suggesting that surgical correction of S2 decreased the cost of energy and facilitated tracheal tree growth and functional recovery of lungs.

Since the patient was under assisted mechanical respiration, the intrathoracic pressure caused by spontaneous respiration was ignored, and the surface of the trachea was assumed to be rigid in the present study. Fluid-structure interaction (FSI) was used to investigate the interaction between the fluid and the tracheal wall in vitro. This technique can be possibly used to study the effects of tracheal compliance on local aerodynamics in the future. The present study focused on a single patient. However, the surgical design using CAD and CFD is indicated for the correction of complex CTS with multiple stenoses.

There are still some limitations of our study; we only compared M2, M3, and M4 in our study, without involving other surgical strategies, such as slide tracheoplasty. The flow pattern of mechanical ventilation will be variable in different ventilation modes. In this pilot aerodynamic study in a patient with CTS, a constant airflow with the volume control of 3 L/min was used to simulate the airflow as the research published in the past. We do agree with the reviewer that comparing the different flow patterns with spontaneous respiration and mechanical ventilation will be a meaningful work. There will be a lot of work to be done in the future.

## 5. Conclusions

The analysis of local aerodynamics using CFD facilitates quantitative evaluation of surgical design for multiple segments of complex CTS. Pressure drops, wall shear stress, airflow distribution, and EL rate are critical elements in the analysis of local aerodynamic features of the trachea. Alterations in the surgical design of the staged correction greatly affect the aerodynamic interaction between multiple stenoses. Multiple segmental CTS should be treated as a comprehensive and interactional lesion. Patient-specific studies using CFD are a potentially noninvasive method to determine the airflow parameters. It minimizes the risk and optimizes the outcomes of staged surgical correction.

## Figures and Tables

**Table 1:** Patient information and CTS classification.

Age (months)	Weight (kg)	Gender	Diagnosis	Clinical severity	Wells classification
5	7.2	Male	CTS PA-Sling LSVS	Severe B	2B

**Table 2:** Mesh information for each model.

Model	M1	M2	M3	M4
Total nodes	217,629	220,892	217,346	218,500
Total elements	783,866	797,068	781,924	792,020

**Table 3:** Maximum value of wall shear stress (WSS) at the regions of stenosis 1 (S1) and stenosis 2 (S2) of each model.

Model	WSS (Pa)	
	S1	S2
M1	9.38	37.82
M2	1.36	47.69
M3	9.61	6.54
M4	1.42	6.40

## Data Availability

The datasets generated during and/or analysed during the current study are available from the corresponding author on reasonable request.
